# *Never Resting Brain:* Simultaneous Representation of Two Alpha Related Processes in Humans

**DOI:** 10.1371/journal.pone.0003984

**Published:** 2008-12-19

**Authors:** Eti Ben-Simon, Ilana Podlipsky, Amos Arieli, Andrey Zhdanov, Talma Hendler

**Affiliations:** 1 Sackler Faculty of Medicine, Tel Aviv University, Tel Aviv, Israel; 2 Functional Brain Center, Wohl Institute for Advanced Imaging, Tel-Aviv Sourasky Medical Center, Tel-Aviv, Israel; 3 Department of Biomedical Engineering, Tel Aviv University, Tel-Aviv, Israel; 4 Neurobiology Department, Weizman Institute, Rehovot, Israel; 5 Department of Psychology, Tel Aviv University, Tel Aviv, Israel; University of Nebraska, United States of America

## Abstract

Brain activity is continuously modulated, even at “rest”. The alpha rhythm (8–12 Hz) has been known as the hallmark of the brain's idle-state. However, it is still debated if the alpha rhythm reflects synchronization in a distributed network or focal generator and whether it occurs spontaneously or is driven by a stimulus. This EEG/fMRI study aimed to explore the source of alpha modulations and their distribution in the resting brain. By serendipity, while computing the individually defined power modulations of the alpha-band, two simultaneously occurring components of these modulations were found. An ‘induced alpha’ that was correlated with the paradigm (eyes open/ eyes closed), and a ‘spontaneous alpha’ that was on-going and unrelated to the paradigm. These alpha components when used as regressors for BOLD activation revealed two segregated activation maps: the ‘induced map’ included left lateral temporal cortical regions and the hippocampus; the ‘spontaneous map’ included prefrontal cortical regions and the thalamus. Our combined fMRI/EEG approach allowed to computationally untangle two parallel patterns of alpha modulations and underpin their anatomical basis in the human brain. These findings suggest that the human alpha rhythm represents at least two simultaneously occurring processes which characterize the ‘resting brain’; one is related to expected change in sensory information, while the other is endogenous and independent of stimulus change.

## Introduction

Since the discovery of Electroencephalography (EEG), attempts have been made to assign a functional meaning to the brain's oscillatory neural activity. The frequency spectrum of scalp recorded EEG has typically been divided into a few bands ranging from delta (less than 3 Hz) to gamma (more than 30 Hz). Each band has been typically attributed to a certain brain state such as the level of consciousness or the degree of cognitive or perceptual activity, respectively [1], [2]. Among the various frequency bands the alpha rhythm (8–12 Hz), received special attention since it was the first oscillation to be identified from scalp recordings. Hans Berger in 1929 found that the alpha power increases during eyes closed especially at rest and decreases with eyes open (i.e. the “Berger effect”) [1]. Subsequently, it has been acknowledged that task engagement such as perceptual judgment or increased attentiveness leads to a decrease in the alpha power [2], [3].

The functional role of alpha has been debated; classically it is considered as the brain's “idle rhythm”, sort of a standby state that allows the system to return more rapidly to goal oriented function when needed [4]. Interestingly, several recent imaging studies have supported the notion of an activated rest state by describing a network of activation that is being diminished during goal oriented tasks relative to no-task [5]–[7]. The idea of alpha as reflecting an idle state was supported by the findings of increased alpha power in posterior electrodes when eyes are closed and in motor cortex when limbs are at rest (also known as the mu rhythm [8]), as well as by studies showing increased alpha rhythm during meditation and other relaxation states [9]. Accordingly, the alpha rhythm was also found to be dominant in states of coma as well as in deep sleep stages [8], [10]. Furthermore, alpha was shown to be negatively correlated with individual arousal levels [11], a fact which might explain the large inter-individual variability in the power and frequency of the alpha rhythm [12], [13]. Yet, the debate over the neural function of alpha rhythm continues with recent theories that assign alpha a more active role in inhibitory control and timing of cortical processing [14], and as being involved in various brain functions such as memory and motor-action [15], [16]. Altogether, although the knowledge on alpha rhythm function is increasing rapidly there are still open issues as for its neural source: is it focal or diffused and what is the basis for its modulations?

Studies in animals suggest that the thalamus is an important source in generation and modulation of cortical alpha rhythms[17] and exhibits a substantial relationship with the neocortex's rhythmic activity [18]. However others, based on animal intracranial recordings, argue that alpha should not be attributed to a specific location but rather to diffuse distributed brain activation since it has been observed in widely separated locations in different brain structures [15], [16]. One way or another it is still unclear if the animal's alpha reflects sufficiently the human's alpha, especially if one considers it as the idle ongoing state of our mental working. Therefore, high spatial resolution studies in humans are essential in order to attain a valid concept regarding the distributed effect of the alpha rhythm.

In humans several EEG studies have pointed to more than a single origin for the alpha rhythm: one which is spontaneous, resulting from distributed sources and unassociated with any external stimulus; and another that is more localized and induced by a stimulus [15], [16], [19]. One drawback of the scientific effort so far in revealing the neural representations of these alpha rhythms is that the majority of studies were done with EEG which suffers from low spatial resolution (∼1 cm). The recent use of combined electrical and cerebral blood flow neuroimaging methods provided the means to explore the alpha rhythm in-vivo in humans at a better spatial resolution than with EEG alone. Furthermore, since the alpha rhythm fluctuates rapidly, simultaneous rather then separate acquisition of these two imaging methods is crucial for a comprehensive view of the alpha correlates. Initial studies applying simultaneous EEG/fMRI recordings in humans revealed distributed BOLD activation correlated to power fluctuations in alpha rhythm. These activations generally included parieto-temporal areas in positive correlation and occipital areas in negative [20]–[22].

This EEG/fMRI study that was originally aimed at exploring the effects of eyes states (i.e. closed or open) on the alpha rhythm, serendipitously revealed two computationally different neural correlates of the alpha rhythm which might underlie different sources of its modulations; induced and spontaneous.

In order to differentiate between induced and spontaneous alpha modulations two components of the overall alpha power changes were computed: one taking into account the induced modulations by eyes states (i.e. close or open), and the other on-going and unrelated to eyes states. By using simultaneous recording of EEG and fMRI we were able to achieve high temporal and spatial resolution. To account for the expected inter-subject variability in alpha ‘finger-print’ we used individually characterized alpha power peak and band [13].

## Methods

### Participants & Study Design

14 healthy volunteers (6 men and 8 women), aged 19–35 (mean 24.8±3.7), signed an informed consent for this study, approved by the Sourasky Medical Center Helsinki committee in Tel Aviv. This approval means that the study was done following the guidelines of the Helsinki Declaration. Subjects were equipped with earphones and asked by means of audio instructions to open and close their eyes every 30 seconds for a total time of 3 minutes. Subjects were told to lie as still as possible and follow the instructions. Sponge cushions were used to minimize head movements.

### EEG acquisition

Continuous EEG data was recorded simultaneously with fMRI acquisition for 200 seconds. EEG was acquired using the MRI-compatible BrainAmp-MR EEG amplifier (Brain Products, Munich, Germany) and the BrainCap electrode cap with sintered Ag/AgCl ring electrodes providing 30 EEG channels, 1 ECG channel, and 1 EOG channel (Falk Minow Services, Herrsching-Breitbrunn, Germany). The electrodes were positioned according to the 10/20 system. The reference electrode was between Fz and Cz. Raw EEG was sampled at 5 kHz and recorded using the Brain Vision Recorder software (Brain Products).

### EEG analysis

### EEG data underwent the following processing stages

#### 1. MR gradient artifacts removal

Artifacts related to the MR gradients were removed from all the EEG datasets using the FASTR algorithm implemented in FMRIB plug-in for EEGLAB, provided by the University of Oxford Centre for Functional MRI of the Brain (FMRIB). Briefly, the FASTR algorithm first corrects for possible minor jitters in the gradients' occurrence delays, and then FASTR computes a template of the artifact based on the slices' average, and subtracts it from the data. Following this process, residual artifacts are reduced using subtraction of Optimal Basis Set (OBS) constructed of first, most meaningful, Principle Components (PCAs) automatically determined from the plot of ordered eigenvalues of the artifacts' matrix.

#### 2. Cardiobalistic artifacts removal

Cardiobalistic artifacts were also removed using the FMRIB plugin, in two stages: a. Detection of QRS events is performed on the ECG channel using combined adaptive thresholding[23] and the Teager energy operator [24], followed by a correction algorithm, which aligns all events and corrects for false positives and negatives. b. Pulse Artifact Removal–The removal of pulse artifacts uses QRS events to subtract an artifact template from the data. This method is similar to the OBS algorithm used to remove the gradient residuals.

After these processing stages the EEG data was downsampled to 250 Hz and underwent a visual inspection of the EOG data for the presence of blinks at the time of instructions, in order to insure that the subjects closed and opened their eyes at those times. This examination led to the exclusion of 2 subjects. Two more subjects were excluded from the analysis due to movements in the scanner which were larger than 1mm. Thus our final analysis included 10 subjects.

#### Individual alpha band calculation

It was previously shown that alpha band frequency varies significantly across subjects[25] therefore it is desirable to estimate alpha band individually for each subject. Our experimental setup induces alpha wave activity facilitating individual characterization of subjects' alpha band. In our analysis we derived the individual subjects' most relevant frequencies from the EEG data with minimal assumptions as to the frequency of the presumed alpha band.

**Figure 1:**
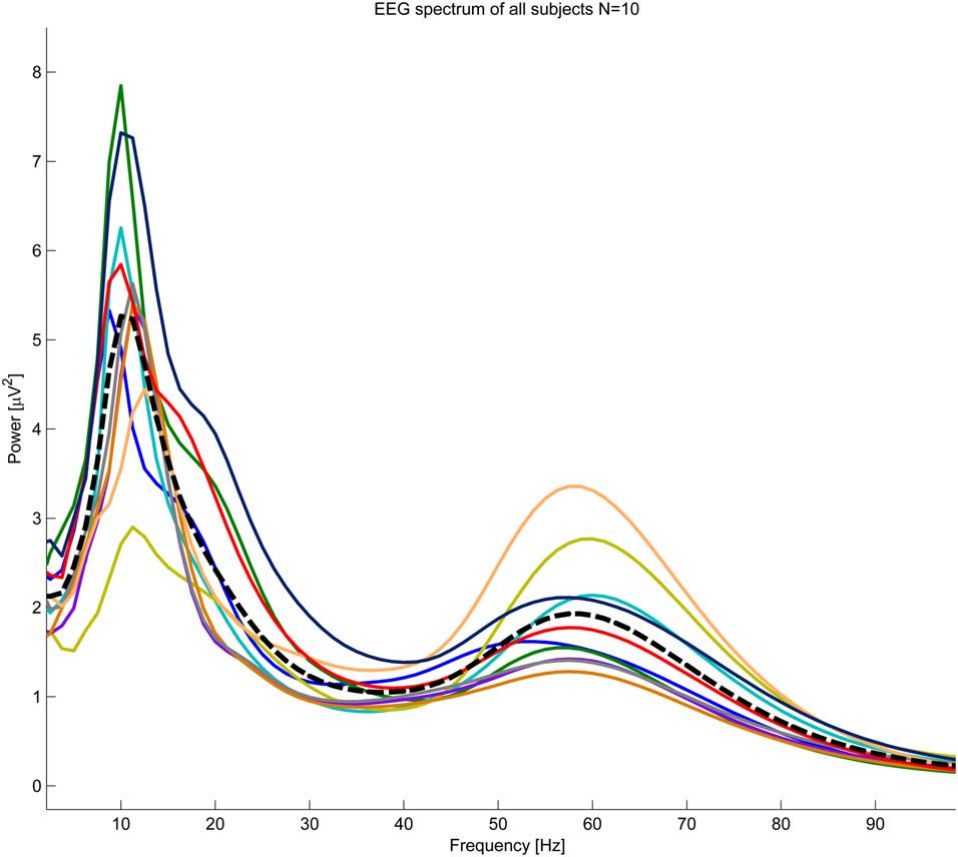
Individual Spectrum plots (n = 10, indicated by different colors). Although the peak power frequency is within conventional alpha band, there is significant inter-subject variability in alpha power and frequency peaks. Average spectrum denoted by a dashed line.

**Figure 2:**
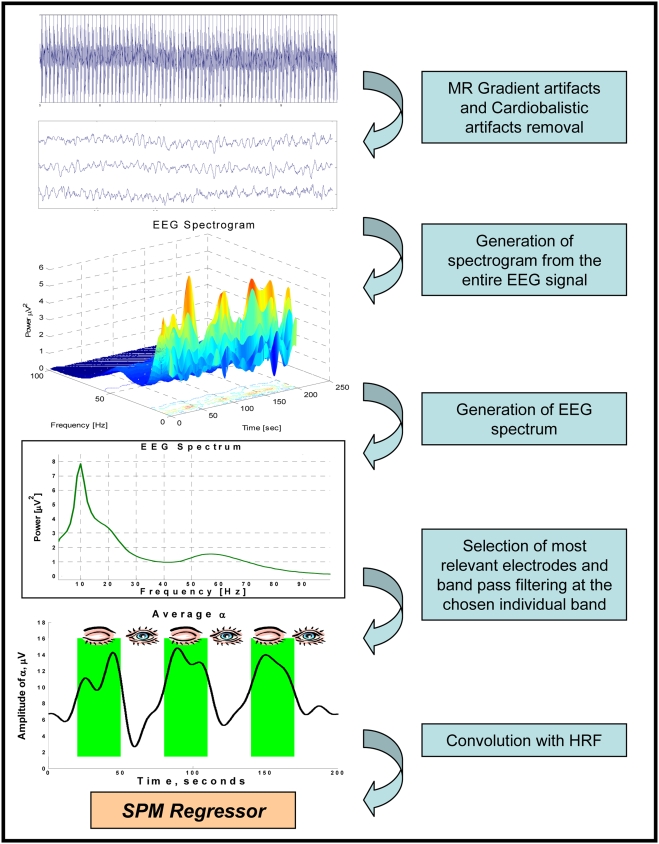
EEG analysis steps. 1. Gradient and cardioballistic artifact removal performed on raw EEG data; 2. Generation of spectrogram from the entire EEG signal; 3. Generation of EEG spectrum by averaging the spectrogram across the time of the experiment (200 sec); 4a. Selection of five electrodes with the highest alpha peak and selection of subject specific alpha band from the EEG spectrum; 4b. Calculation of instantaneous alpha amplitude throughout the experiment, at the chosen electrodes by means of band pass filtering at the chosen band and Hillbert transform; 5. Calculation of a regressor for fMRI analysis by convolution of the instantaneous alpha amplitude with HRF.

The EEG signal was expected to have, on average, high amplitude at the conventional alpha band frequencies (see Fig. 1). The individual alpha band was taken as the frequency band containing the highest energy of the signal across the experiment. The first step of the individual alpha band estimation was to calculate instantaneous power at each frequency of the EEG, using Stockwell transform[26] with frequency resolution of 1.25 Hz and time resolution of 1/250 sec (using Matlab 7.0.4, Mathworks Inc). The resulting instantaneous power was then averaged across the whole experiment at each electrode (see EEG spectrogram at Fig. 2).

In the search for the individual alpha band, the EEG spectrum was examined at each electrode, and a frequency (lower than 20 Hz) with the highest power was chosen as the alpha peak. Finally the five electrodes with the highest peaks were taken for further analysis. The distribution of alpha power across electrodes reveals that the greatest power is localized in occipital regions. Accordingly, Most of the chosen electrodes per subjects were located in the occipital-temporal area (see supplementary Table S1 for a list of the chosen electrodes). The individual subjects' alpha band, across the chosen electrodes, was taken to be the alpha peak frequency ±1.25 Hz. This band width covered most of the alpha peak for most subjects (see supplementary Table S1 for different alpha characteristics of all subjects).

#### Continuous regressor generation

for each subject, the original EEG signal of the five chosen electrodes was band pass filtered to the individual subjects' alpha band. At each of these electrodes instantaneous amplitude of the resulting signal was calculated by means of Hilbert transform[27] and low pass filtered at 0.05 Hz to adopt it to the fMRI temporal resolution. Resulting signals were averaged across the five electrodes. The average signal was taken as the individual regressor for the fMRI analysis of each subject. For a summary of EEG analysis and regressor generation see Figure 2.

### fMRI acquisition

Imaging was performed on a 3 T GE scanner (GE, Milwaukee, WI, USA). All images were acquired using a standard head coil. The scanning session included conventional anatomical MR images (T1-WI, T2-WI, T2-FLAIR), 3D spoiled gradient echo (SPGR) sequence (FOV- 250 mm, matrix size- 256×256, voxel size 0.98×0.98×1) and functional T2*-weighted images (FOV = 200 mm, matrix size- 64×64, voxel size-3×3×4, TR/TE = 2000/35, Slice thickness = 4 mm, 30 axial slices without gap).

### fMRI analysis

SPM2 software (http://www.fil.ion.ucl.ac.uk/spm) was used for image preprocessing and voxel-based statistical analysis. First 20 seconds of data were discarded to allow steady state magnetization. Functional images were realigned to the first scan and normalized into standard MNI space. Spatial smoothing was performed using a Gaussian kernel (FWHM = 4 mm) and the signal was high-pass filtered at 1/128 s. To correlate the fMRI with the EEG data, the individual alpha time course (see EEG analysis) was used as a regressor in the design matrix, which also included a mean term.

Since an eyes open eyes closed paradigm induces alpha wave modulation it was necessary to determine whether the functional network derived from the EEG alpha regressor differs from the functional network revealed by the paradigm conditions. For that purpose an additional analysis was performed in which the design matrix was comprised only of the eyes open eyes closed conditions. The alpha regressor and the paradigm are strongly correlated, therefore the two functional networks, revealed by each of them, were largely similar. Hence, the next step of the analysis was to calculate a paradigm-independent alpha regressor by introducing a confounding covariate of eyes open eyes closed conditions into the design matrix. This procedure removes the part of the alpha curve linearly explainable by the paradigm (the induced alpha part), and leaves the spontaneous fluctuations of alpha power occurring within conditions. For group analysis, a random-effects model was applied, and statistical inferences were considered significant at p<0.02, uncorrected.

## Results

### Individually determined EEG spectrum

Overall EEG revealed the expected peak of alpha in the dataset ranging from 7.5 to 12.25 Hz among the different subjects (see supplementary Table S1 for more details) and another peak is present at a frequency range that corresponds with gamma (40–80 Hz). A possible correlation between gamma and alpha time courses was examined over the course of the data analysis and no significant correlation was found. However, here we only present results that correspond to the alpha peak. Figure 1 denotes the individual spectra presented by different colors and the averaged peak shown in black. The spectra clearly demonstrate the relatively large variance among individuals in overall alpha power and frequency band in the data set.

### Bold activation in relation to induced alpha modulations

**Figure 3:**
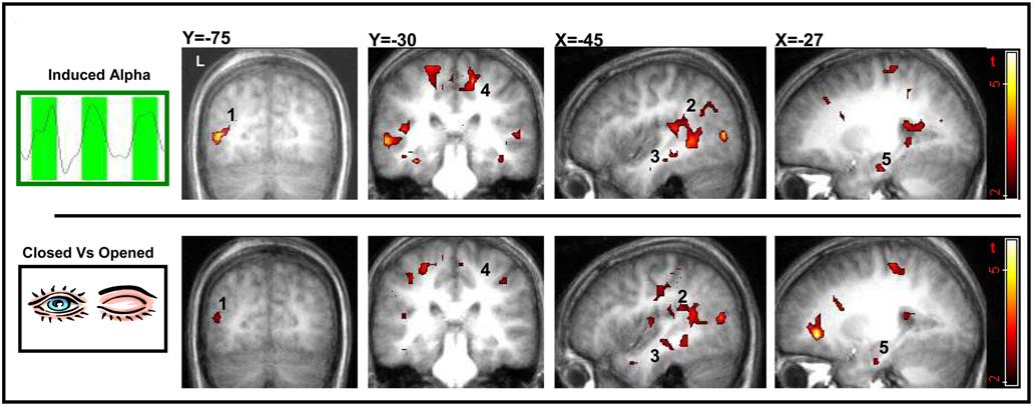
Induced alpha and paradigm related BOLD activation maps. Different slice views of BOLD activation group maps (n = 10 random effect, p<0.02 uncorrected, min 15 voxels). Top row maps obtained by individual regressors of the induced alpha component. Low row maps obtained directly by the paradigm conditions (i.e. eyes closed Vs eyes open). Note the similarities between the two maps. Areas of main activation are denoted by numbers: L Middle Occipital Cortex (1), L Superior Temporal Sulcus (2), L Middle Temporal Gyrus (3), L Supplementary Motor Area (4), L Hippocampus (5).

We first examined the effect of induced alpha modulation on BOLD signal change, taking into account eyes state. Figure 3 (top row) demonstrates the corresponding alpha based group activation map (n = 10, p<0.02 random effects, uncorrected). A positive correlation between the BOLD signal and the individual alpha regressor revealed a distributed network including activation in posterior and anterior Superior Temporal Sulcus (STS), more prominent on the left; the Supplementary Motor Area (SMA) and bilateral anterior Hippocampus (see supplementary Table S2). We then examined the effect of the paradigm (eyes open Vs eyes closed) on the BOLD signal, disregarding alpha. Figure 3 (bottom row) demonstrates the paradigm based group activation map (n = 10, p<0.02 random effects, uncorrected). From comparing the activation maps in upper and lower rows one can clearly see the large similarity between the paradigm based and the induced-alpha, bold activation maps, implying that the paradigm activations masked over alpha based activations.

### BOLD activation in relation to spontaneous alpha modulation

**Figure 4:**
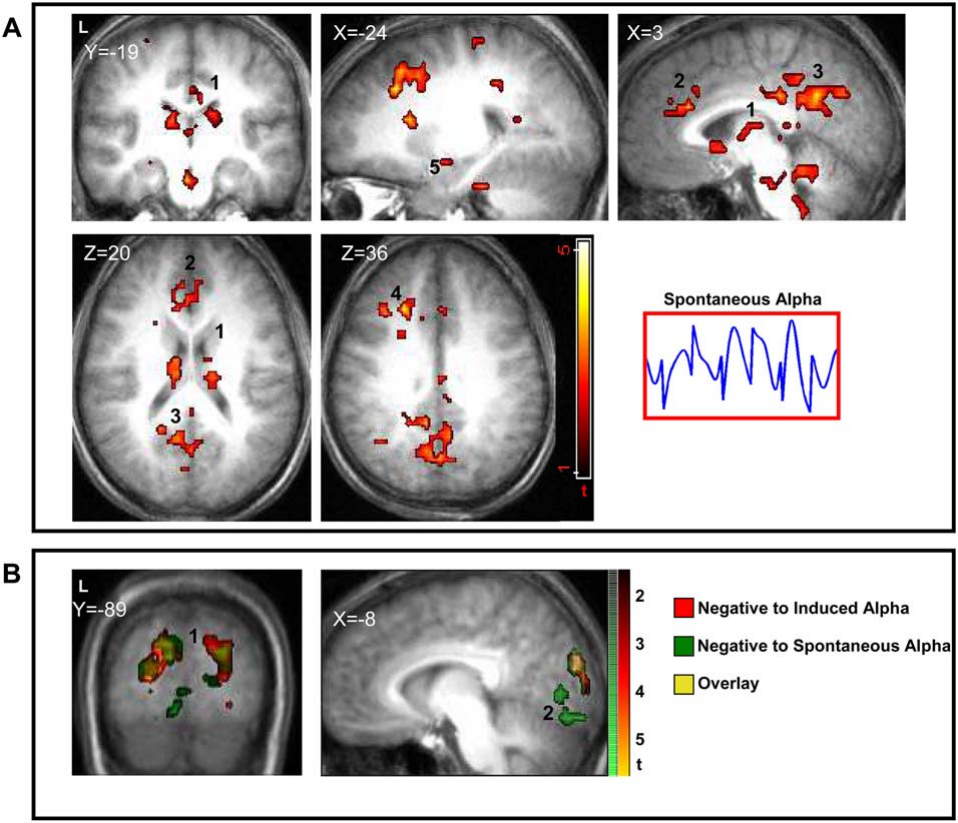
Spontaneous and negative alpha BOLD activation maps. A. Spontaneous alpha BOLD activation maps: Different slice views of BOLD activation group maps (n = 10 random effect, p<0.02 uncorrected, min 15 voxels) obtained by individual regressors of the spontaneous alpha component. Areas of activation are denoted by numbers: Dorso Medial Thalamus (1), Medial Prefrontal Cortex (2), Retrosplenial Cortex (3), Dorso Lateral Prefrontal Cortex (4), Amygdala (5). B. Negative alpha BOLD activation maps: Different slice views of BOLD activation group maps (n = 10 random effect, p<0.02 uncorrected, min 15 voxels) obtained by negative correlation to individual regressors of induced and spontaneous alpha components (red and green respectively). As expected negative correlation in both networks reveal predominantly visual areas. Areas of activation are denoted by numbers: primary Visual Cortex (1), high order visual areas (2).

**Figure 5:**
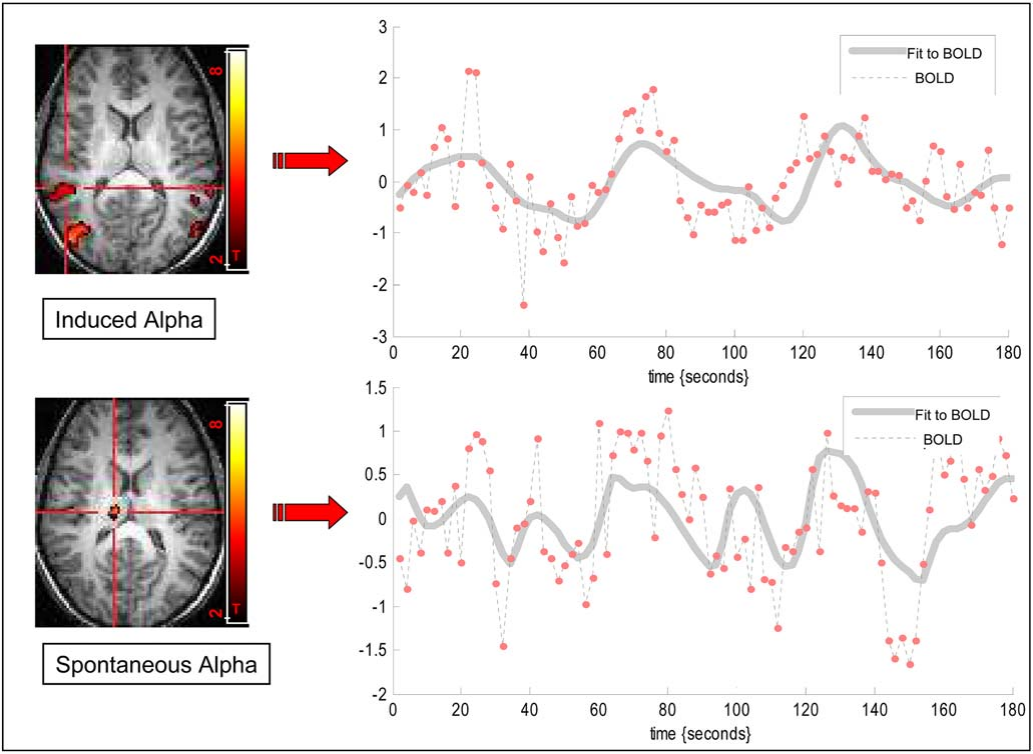
BOLD time course related to spontaneous and induced alpha networks. BOLD time course of a single subject was extracted from induced alpha region of interest (ROI) in left STS (top, P<0.0001) and from the spontaneous alpha network in the thalamus (bottom, P<0.0001). The dashed line denotes the measured BOLD time course and the grey line denotes best fit to BOLD oscillations. It is evident that the BOLD time course extracted from the induced alpha network corresponds to the paradigm based alpha modulation while the time course based on spontaneous oscillations corresponds to the on-going alpha modulation.

In order to reveal possible alpha modulation that is unrelated to eyes states we applied an analysis that aimed to computationally eliminate the effect of the paradigm on the alpha variation which resulted in the spontaneous alpha regressor. A positive correlation between the BOLD signal and the spontaneous alpha regressor revealed a distributed network including the Dorso Latreal Pre-Frontal Cortex, Caudate and Thalamic nuclei. This network is demonstrated in Figure 4 and in supplementary Table S3. A comparison of BOLD signal extracted from ROI at the spontaneous alpha network Vs ROI from induced alpha network is demonstrated in Figure 5.

### Negative correlation with alpha rhythm

Negative correlation with the individual alpha regressor, in both induced and spontaneous networks, reveals predominantly low level visual areas in the occipital lobe (see Fig. 4B, supplementary Table S2 and S3).

## Discussion

The main finding of this study is that the alpha rhythm detected in scalp EEG recordings was associated with two concurrent modulations that yielded segregated distributions of BOLD activation as revealed by the simultaneous fMRI: one that was closely linked to the difference between eyes open and eyes closed states and the other that was largely independent of these changing states (see Fig. 5). This finding emanates from our approach of computationally separating the spontaneous from the induced alpha modulations. Possibly, these two simultaneous organizations of alpha modulation have not been demonstrated before as other EEG studies combined with cerebral blood flow imaging have mainly concentrated on eyes-closed rest state thus revealing mainly the spontaneous alpha network [22], [28]. Indeed, these studies report on alpha related BOLD activation that closely resembles the spontaneous alpha distribution in our study. In one attempt to measure the induced alpha rhythm by EEG/fMRI the large “Berger effect” due to alternating eyes states seemed to mask the possible existing spontaneous modulations [28].

Negative correlation to both induced and spontaneous alpha modulations were found predominantly in low-level visual areas in the occipital cortex (see Fig. 4B). This is in accordance with several fMRI and PET studies that showed negative alpha related correlation in occipital regions, mainly in primary visual areas [20]–[22], [28]. Together these studies suggest that an increase in alpha power is accompanied by a decrease in overall activation in primary and secondary visual areas. Furthermore, EEG studies showed that desynchronization of alpha rhythm in response to visual input represents overall increased activation in the visual system [11]. In accordance with this view the occipital cortex has been indicated by several EEG and MEG studies as an alpha generator region[29], [30] or, from the viewpoint of distributed alpha generators, as an area in which the alpha generators are dense [15]. However, since gamma activity was shown to be involved in integrated visual processing [31], one cannot preclude completely the possibility that the observed alpha related deactivation in the current study is also accompanied by increased gamma synchronization.

### Activation related to spontaneous modulation of the alpha rhythm

In addition to distributed cortical activation, the spontaneous alpha modulations yielded a significant BOLD activation in the thalamus (see Fig. 4A), traditionally thought of as the main source of the alpha rhythm in the mammalian brain [17]. This conclusion was based on cellular recordings in animals exhibiting thalamic oscillation in the 8–12 Hz, and further studies which revealed a substantial relationship between rhythmic thalamic activity and distributed cortical activity [18], [32].

Neuroimaging studies have been inconsistent as to the thalamic origin of alpha. For example, a PET/EEG study on humans at rest described a positive correlation between alpha power and metabolic activity in bilateral thalamic nuclei [33], in accordance, more recent human EEG/fMRI studies also revealed a positive correlation between BOLD activation in the thalamus and the power of scalp recorded alpha rhythm [20]–[22]. In contrast to these results an EEG/fMRI rest study by Laufs et al[28] was inconclusive as to the role of thalamic nuclei in the generation of alpha rhythm, showing no thalamus alpha correlation. This difference in whole-brain combined measures might be related to the existence of more than one neural component of the alpha rhythm and to its sensitivity to individual's mental state.

Interestingly, in our study the thalamus was not part of the induced alpha network but rather of the spontaneous alpha network, suggests its involvement in the on-going stimulus independent modulations of alpha. It is therefore possible that the thalamus subserves the mechanism of alpha generation that concords with its role as an ongoing pace-maker. It was previously proposed that spontaneous modulations of the alpha rhythm, like a central pace-maker, might coordinate rhythmical activity in widely distributed cortical areas by synchronization [31], serving as a main contributor to the brain's “self resonance” in on-going activity [15].

In contrast to the common view of the thalamus as a single alpha generator, some data point to the possibility that alpha rhythm might have non-thalamic sources. For example, in dogs it was shown that even after computationally eliminating thalamic activity the coherence of cortical alpha rhythms remains, albeit decreased [32]. Similarly, in humans, bilateral thalamic lesions did not abolish posterior alpha rhythm in eyes closed state [34]. These results correspond to a claim made lately by several researches that the alpha rhythm is driven by several neural generators [15], [16].

### Alpha rhythm and the ‘default brain’

It is noteworthy that in our study the BOLD activation network derived from the spontaneous alpha partly overlaps with the commonly described BOLD deactivation during goal directed tasks, also known as the “default brain” (e.g. see [5]–[7], [35] and for comparison Figure 4). This overlap corresponds with early ideas that the alpha rhythm reflects an ‘idle state’ of the brain [4]. Intriguingly, in a recent fMRI study the typical “default brain” activation was not affected by eyes position and was similar for eyes open and eyes closed. This finding lead to the assumption that the deactivation network is distinct from the alpha related network[6] since the latter changes with eyes state. However, our study suggests that any relation of alpha modulation to the “default brain” network is possible only if it is differentiated from the periodic effect of eyes state. A recent imaging study further supports this claim by showing computationally six spatiotemporal distinct rest related BOLD activations. A correlation of the various EEG bands to these BOLD networks revealed a strong relationship of alpha power with three networks, one of which corresponds to most regions of the commonly defined “default brain”. Intriguingly, this network also exhibited significant evidence of thalmo-cortical connectivity[36] similar to the spontaneous alpha network in the current study.

### Activation related to induced modulation of the alpha rhythm

The ‘induced alpha’ network exhibits distributed BOLD activation in the SMA, STS, Hippocampus and Parahippocampal gyrus. Not surprisingly the induced alpha network largely overlaps with the paradigm driven BOLD activation to eyes closed versus eyes open (see Fig. 3 for comparison). Accordingly, the induced network resembles the findings of a recent fMRI rest study[37] in its eyes closed Vs eyes open contrast. It therefore can be concluded that the activation related to the induced alpha modulation closely corresponds to alternation in eyes states possibly relating to changes in visual input. Another suggestion for sensory related alpha generator comes from an EEG/PET study showing a significant correlation with the alpha rhythm during eyes closed in two areas of the brain: the thalamus and the STS [38]. Here we show that these activations derived from two separated components of the alpha rhythm, the thalamus from the spontaneous alpha while the STS from the induced alpha modulation.

The hippocampus was also part of the induced alpha network. This result is compatible with animal recordings in the hippocampus showing enhanced alpha signals with increased sensory stimulation [39]. Since different alpha frequencies were recorded depending on the sensory modality (visual vs. auditory stimuli) it was further suggested that the hippocampus serves a gating mechanism for alpha signals [15], [16], modulating sensory input.

The induced alpha BOLD activations were largely lateralized to the left hemisphere (see supplementary Table S2). This alpha related functional asymmetry was previously reported by scalp recorded EEG in occipital and temporal regions of awaked subjects at rest, both with eyes open and closed [10]. Creutzfeldt [40] suggested that there are distinct alpha generators in each hemisphere which share a common control mechanism; according to this view it might be suggested that the left generator is more involved in processes of sensory induced modulations.

### Methodological considerations

Several limitations of our study should be noted. 1) The baseline condition alternating between the eyes open eyes closed condition might have hampered the sensitivity of our findings. It could be that these networks are especially attuned not to one state or the other but rather to the actual transition from one state to another. 2) We only studied visual related alpha modulations thus we cannot generalize with regard to other modalities that might generate rhythms of similar frequency bands such as action or auditions. 3) Different modulation states might exist in other frequency bands mediating different functions in the brain. This study focused on the alpha band alone. 4) Finally, the functional characterization of each alpha related network requires further studies that will manipulate each network separately by stimuli, task or pharmacological conditions.

### In summary

To our knowledge this is the first human brain study to demonstrate two spatially segregated yet simultaneously active networks associated with alpha rhythm modulations: one is spontaneous and on-going and the other is induced and periodic. These findings support the claim made already by Walter Grey (1950, quoted by Basar [15]) that the scalp recorded alpha is the end product of many alpha rhythms that are spatially averaged over the scalp. A similar idea was expressed more recently by Nunez et al[1] saying: “the human alpha band contains multiple rhythms that apparently interact to varying degrees in different brain states” (quoted from [1]). Here by using simultaneous acquisition of EEG/fMRI we were able to disentangle at least two of these concurrent rhythmic modulations in the intact human brain; the induced and the spontaneous. The notion of an alpha rhythm that is comprised of two components, might correspond to the suggestion of two endogenous processes of the “ resting brain”; one which is tuned outward and is periodic, the other which is focused inward and is persistent [35].

## Supporting Information

Table S1 Individual alpha characteristics of studied subjects (n = 10)
(0.06 MB DOC)

Table S2 Clusters of BOLD activation significantly correlated with induced alpha
(0.07 MB DOC)

Table S3 Clusters of BOLD activation significantly correlated with spontaneous alpha
(0.06 MB DOC)

## References

[1] Nunez PL, Wingeier BM, Silberstein RB (2001). Spatial-temporal structures of human alpha rhythms: theory, microcurrent sources, multiscale measurements, and global binding of local networks.. Hum Brain Mapp.

[2] Niedermeyer E, Silva FLd (2005). Electroencephalography: Basic Principles, Clinical Applications, and Related Fields.

[3] Pfurtscheller G, Silva FHLd (1999). Event-related EEG/MEG synchronization and desynchronization: basic principles.. Clin Neurophysiol.

[4] Adrian ED, Matthews BH (1934). The interpretation of potential waves in the cortex.. J Physiol.

[5] Raichle ME, MacLeod AM, Snyder AZ, Powers WJ, Gusnard DA (2001). A default mode of brain function.. Proc Natl Acad Sci U S A.

[6] Greicius MD, Krasnow B, Reiss AL, Menon V (2003). Functional connectivity in the resting brain: a network analysis of the default mode hypothesis.. Proc Natl Acad Sci U S A.

[7] Gusnard DA, Raichle ME, Raichle ME (2001). Searching for a baseline: functional imaging and the resting human brain.. Nat Rev Neurosci.

[8] Niedermeyer E (1997). Alpha rhythms as physiological and abnormal phenomena.. Int J Psychophysiol.

[9] Travis F, Wallace RK (1999). Autonomic and EEG patterns during eyes-closed rest and transcendental meditation (TM) practice: the basis for a neural model of TM practice.. Conscious Cogn.

[10] Benca RM, Obermeyer WH, Larson CL, Yun B, Dolski I (1999). EEG alpha power and alpha power asymmetry in sleep and wakefulness.. Psychophysiology.

[11] Barry RJ, Clarke AR, Johnstone SJ, Magee CA, Rushby JA (2007). EEG differences between eyes-closed and eyes-open resting conditions.. Clin Neurophysiol.

[12] Goncalves SI, de Munck JC, Pouwels PJ, Schoonhoven R, Kuijer JP (2006). Correlating the alpha rhythm to BOLD using simultaneous EEG/fMRI: inter-subject variability.. Neuroimage.

[13] Klimesch W (1999). EEG alpha and theta oscillations reflect cognitive and memory performance: a review and analysis.. Brain Res Brain Res Rev.

[14] Klimesch W, Sauseng P, Hanslmayr S (2007). EEG alpha oscillations: the inhibition-timing hypothesis.. Brain Res Rev.

[15] Basar E, Schurmann M, Basar-Eroglu C, Karakas S (1997). Alpha oscillations in brain functioning: an integrative theory.. Int J Psychophysiol.

[16] Schurmann M, Basar E (2001). Functional aspects of alpha oscillations in the EEG.. Int J Psychophysiol.

[17] Andersen P, Andersson SA (1968). Physiological Basis of the Alpha Rhythm.

[18] Da-Silva LF, Lierop THv, Schrijer CF, Leeuwen WSv (1973). Organization of thalamic and cortical alpha rhythms: spectra and coherences.. Electroencephalogr Clin Neurophysiol.

[19] Basar E, Basar-Eroglu C, Karakas S, Schurmann M (2001). Gamma, alpha, delta, and theta oscillations govern cognitive processes.. Int J Psychophysiol.

[20] Sadato N, Nakamura S, Oohashi T, Nishina E, Fuwamoto Y (1998). Neural networks for generation and suppression of alpha rhythm: a PET study.. Neuroreport.

[21] Moosmann M, Ritter P, Krastel I, Brink A, Thees S (2003). Correlates of alpha rhythm in functional magnetic resonance imaging and near infrared spectroscopy.. Neuroimage.

[22] Goldman RI, Stern JM, Engel J, Cohen MS (2002). Simultaneous EEG and fMRI of the alpha rhythm.. Neuroreport.

[23] Christov II (2004). Real time electrocardiogram QRS detection using combined adaptive threshold.. Biomed Eng Online.

[24] Kim kh, Yoon Hw, Park Hw (2004). Improved ballistocardiac artifact removal from the electroencephalogram recored in FMRI.. J Neourosience Methods.

[25] Miller R (2000). Time and the Brain.

[26] Stockwell RG, Mansinha L, Lowe RP (1996). Localization of the complex spectrum: the S transform.. Signal Processing, IEEE Transactions on.

[27] Le Van Quyen M, Foucher J, Lachaux J, Rodriguez E, Lutz A (2001). Comparison of Hilbert transform and wavelet methods for the analysis of neuronal synchrony.. J Neurosci Methods.

[28] Laufs H, Kleinschmidt A, Beyerle A, Eger E, Salek-Haddadi A (2003). EEG-correlated fMRI of human alpha activity.. Neuroimage.

[29] Chapman RM, Ilmoniemi RJ, Barbanera S, Romani GL (1984). Selective localization of alpha brain activity with neuromagnetic measurements.. Electroencephalogr Clin Neurophysiol.

[30] Nunez PL, Srinivasan R (2006). Electric Fields of the Brain: The Neurophysics of EEG.

[31] Stein Av, Sarnthein J (2000). Different frequencies for different scales of cortical integration: from local gamma to long range alpha/theta synchronization.. Int JPsychophysiology.

[32] Da-Silva L, Vos JE, Mooibroek J, Rotterdam AV (1980). Relative contributions of intracortical and thalamo-cortical processes in the generation of alpha rhythms, revealed by partial coherence analysis.. Electroencephalogr Clin Neurophysiol.

[33] Schreckenberger M, Lange-Asschenfeldt C, Lochmann M, Mann K, Siessmeier T (2004). The thalamus as the generator and modulator of EEG alpha rhythm: a combined PET/EEG study with lorazepam challenge in humans.. Neuroimage.

[34] Yazawa S, Kawasaki S, Kanemaru A, Kuratsuwa Y, Yabuoshi R (2001). Bilateral paramedian thalamo-midbrain infarction showing electroencephalographic alpha activity.. Intern Med.

[35] Fox MD, Raichle ME (2007). Spontaneous fluctuations in brain activity observed with functional magnetic resonance imaging.. Nat Rev Neurosci.

[36] Mantini D, Perrucci MG, Del Gratta C, Romani GL, Corbetta M (2007). Electrophysiological signatures of resting state networks in the human brain.. Proc Natl Acad Sci U S A.

[37] Marx E, Stephan T, Nolte A, Deutschlander A, Seelos KC (2003). Eye closure in darkness animates sensory systems.. Neuroimage.

[38] Larson CL, Davidson RJ, Abercrombie HC, Ward RT, Schaefer SM (1998). Relations between PET-derived measures of thalamic glucose metabolism and EEG alpha power.. Psychophysiology.

[39] Schurmann M, Demiralp T, Basar E, Basar Eroglu C (2000). Electroencephalogram alpha (8–15 Hz) responses to visual stimuli in cat cortex, thalamus, and hippocampus: a distributed alpha network?. Neurosci Lett.

[40] Creutzfeldt OD, Creutzfeldt M (1995). Chapter 5: Spontaneous and evoked cortical potentials and related neuronal events.. Cortex Cerebri: In: Performance, Structural and Functional Organisation of the Cortex.

