# Supplementary material for: Never Resting Brain: Simultaneous Representation of Two Alpha Related Processes in Humans
Source: PLoS One. 2008 Dec 19;3(12):e3984. doi: 10.1371/journal.pone.0003984 (PMC2602982; doi:10.1371/journal.pone.0003984)
Supplement: Table S1 — Individual alpha characteristics of studied subjects (n = 10) (0.06 MB DOC) [file pone.0003984.s001.doc]

***Supplementary Table S1:***

***Individual alpha characteristics of studied subjects (n=10).***

| **Subject** | **Age (years)** | **Individual Alpha band (Hz)** | | **Chosen Electrodes** | | | | |
| --- | --- | --- | --- | --- | --- | --- | --- | --- |
|  |  |  |  |  |  |  |  |  |
| 1 | 19 | 8.75 | 11.25 | TP10 | TP9 | P7 | O1 | P8 |
|  |  |  |  |  |  |  |  |  |
| 2 | 24 | 11 | 13.5 | O1 | O2 | Oz | TP9 | P7 |
|  |  |  |  |  |  |  |  |  |
| 3 | 25 | 8 | 10.5 | P8 | TP10 | O2 | P7 | TP9 |
|  |  |  |  |  |  |  |  |  |
| 4 | 22 | 8.75 | 11.25 | P7 | Oz | P8 | O1 | O2 |
|  |  |  |  |  |  |  |  |  |
| 5 | 24 | 8.75 | 11.25 | O2 | O1 | P8 | P7 | Oz |
|  |  |  |  |  |  |  |  |  |
| 6 | 24 | 9.25 | 11.75 | P8 | TP9 | P7 | TP10 | O2 |
|  |  |  |  |  |  |  |  |  |
| 7 | 23 | 8.75 | 11.25 | F8 | P7 | O1 | TP9 | TP10 |
|  |  |  |  |  |  |  |  |  |
| 8 | 23 | 11.5 | 13.75 | O1 | O2 | Oz | TP10 | P8 |
|  |  |  |  |  |  |  |  |  |
| 9 | 23 | 7.5 | 10 | P7 | TP9 | O1 | TP10 | P8 |
|  |  |  |  |  |  |  |  |  |
| 10 | 25 | 10 | 12.5 | FC1 | CP1 | O1 | O2 | Oz |
|  |  |  |  |  |  |  |  |  |
| ***AVG*** | 23.2 | 9.2 | 11.7 |  | | | | |
| ***STED*** | 1.75 | 1.2 | 1.2 |
