# Supplementary material for: Never Resting Brain: Simultaneous Representation of Two Alpha Related Processes in Humans
Source: PLoS One. 2008 Dec 19;3(12):e3984. doi: 10.1371/journal.pone.0003984 (PMC2602982; doi:10.1371/journal.pone.0003984)
Supplement: Table S2 — Clusters of BOLD activation significantly correlated with induced alpha (0.07 MB DOC) [file pone.0003984.s002.doc]

***Supplementary Table S2:***

***Clusters of BOLD activation significantly correlated with induced alpha***

**Significant BOLD activation clusters that were correlated with the induced component of the alpha regressor (random effects, n=10, P<0.009, uncorrected, min 3 voxels).**

| **Anatomical region** | **Side** | **Cluster size** | **Voxel P (unc)** | **Voxel *T* value** | **MNI Coordinates** | | |
| --- | --- | --- | --- | --- | --- | --- | --- |
|  |  |  |  |  |  |  |  |
| ***Positive correlation*** |  |  |  |  | ***x*** | ***y*** | ***z*** |
| Mid Frontal Cortex | **L** | 6 | 0.007 | 3.035 | -24 | 30 | 32 |
| Supplementary Motor Area | R | 40 | 0.001 | 4.440 | 9 | -21 | 60 |
| Mid Cingulate | **L** | 7 | 0.001 | 4.220 | -6 | -9 | 44 |
| Temporal Pole | R | 12 | 0.003 | 3.648 | 54 | -9 | -20 |
| Superior Temporal Sulcus | **L** | 12 | 0.002 | 3.809 | -45 | -30 | 12 |
| Superior Temporal Sulcus | R | 9 | 0.003 | 3.644 | 48 | -48 | 4 |
| Mid Temporal Cortex | **L** | 7 | 0.004 | 3.440 | -45 | -30 | -12 |
| Mid Temporal Cortex | R | 5 | 0.005 | 3.220 | 39 | -30 | -16 |
| Angular gyrus | **L** | 11 | 0.003 | 3.575 | -39 | -57 | 32 |
| Mid Occipital Cortex | **L** | 17 | 0.000 | 5.375 | -45 | -75 | 8 |
| Hippocampus/Para hippocampal gyrus | **L** | 6 | 0.002 | 3.742 | -27 | -18 | -24 |
| Hippocampus/Para hippocampal gyrus | R | 8 | 0.001 | 4.270 | 24 | -18 | -24 |
| Caudate | **L** | 6 | 0.001 | 4.200 | -18 | 15 | 16 |
| Vermis |  | 27 | 0.001 | 4.578 | -6 | -45 | -20 |
|  |  |  |  |  |  |  |  |
| ***Negative correlation*** |  |  |  |  |  |  |  |
| Inferior Frontal gyrus | R | 9 | 0.000 | 5.143 | 54 | 27 | 20 |
| Anterior Insula | R | 4 | 0.001 | 4.184 | 48 | 6 | 0 |
| Lingual gyrus | R | 12 | 0.004 | 3.470 | 12 | -81 | 12 |
| Calcarine | **L** | 88 | 0.001 | 4.330 | -18 | -99 | 4 |
| Calcarine | R | 87 | 0.000 | 6.290 | 24 | -99 | 12 |
| Hippocampus/Para hippocampal gyrus | R | 7 | 0.005 | 3.241 | 21 | -27 | -16 |
