# Supplementary material for: Never Resting Brain: Simultaneous Representation of Two Alpha Related Processes in Humans
Source: PLoS One. 2008 Dec 19;3(12):e3984. doi: 10.1371/journal.pone.0003984 (PMC2602982; doi:10.1371/journal.pone.0003984)
Supplement: Table S3 — Clusters of BOLD activation significantly correlated with spontaneous alpha (0.06 MB DOC) [file pone.0003984.s003.doc]

***Supplementary Table S3:***

***Clusters of BOLD activation significantly correlated with spontaneous alpha***

**Significant BOLD activation clusters that were correlated with the spontaneous component of the alpha regressor (random effects, n=10, P<0.009, uncorrected, min 3 voxels).**

| **Anatomical region** | **Side** | **Cluster size** | **Voxel P (unc)** | **Voxel *T* value** | **MNI Coordinates** | | |
| --- | --- | --- | --- | --- | --- | --- | --- |
|  |  |  |  |  |  |  |  |
| **Positive correlation** |  |  |  |  | ***x*** | ***y*** | ***z*** |
| Dorso Lateral Pre Frontal Cortex | **L** | 32 | 0.001 | 4.432 | -24 | 27 | 36 |
| Anterior Insula | **L** | 4 | 0.003 | 3.664 | -33 | 15 | -16 |
| Mid Cingulate Cortex | **L** | 29 | 0.002 | 3.955 | -6 | 30 | 24 |
| Mid Cingulate Cortex | R | 29 | 0.002 | 3.923 | 3 | 30 | 24 |
| Ventral Inferior Parietal Cortex | **L** | 10 | 0.004 | 3.399 | -42 | -63 | 32 |
| Retrosplenial Cortex |  | 12 | 0.002 | 3.780 | -3 | -27 | 32 |
| Precuneus |  | 29 | 0.001 | 4.176 | 3 | -54 | 32 |
| Thalamus | **L** | 16 | 0.004 | 3.462 | -9 | -15 | 20 |
| Thalamus | R | 7 | 0.002 | 3.697 | 15 | -18 | 16 |
| Amygdala | **L** | 4 | 0.002 | 3.767 | -18 | -6 | -8 |
| Caudate | **L** | 16 | 0.004 | 3.410 | -9 | -6 | 20 |
| Caudate | R | 8 | 0.004 | 3.392 | 15 | 24 | 0 |
| Vermis |  | 36 | 0.000 | 5.097 | -3 | -15 | -24 |
|  |  |  |  |  |  |  |  |
| **Negative correlation** |  |  |  |  |  |  |  |
| Calcarine | **L** | 76 | 0.000 | 4.887 | -18 | -87 | 28 |
| Calcarine | R | 70 | 0.000 | 5.318 | 18 | -93 | 24 |
